# The C-X-C Motif Chemokine Ligand 1 Sustains Breast Cancer Stem Cell Self-Renewal and Promotes Tumor Progression and Immune Escape Programs

**DOI:** 10.3389/fcell.2021.689286

**Published:** 2021-06-14

**Authors:** Stefania Livia Ciummo, Luigi D’Antonio, Carlo Sorrentino, Cristiano Fieni, Paola Lanuti, Giorgio Stassi, Matilde Todaro, Emma Di Carlo

**Affiliations:** ^1^Department of Medicine and Sciences of Aging, “G. d’Annunzio” University, Chieti, Italy; ^2^Anatomic Pathology and Immuno-Oncology Unit, Center for Advanced Studies and Technology (CAST), “G. d’Annunzio” University, Chieti, Italy; ^3^Department of Surgical, Oncological and Stomatological Sciences (DICHIRONS), University of Palermo, Palermo, Italy; ^4^Department of Health Promotion Sciences, Internal Medicine and Medical Specialties (PROMISE), University of Palermo, Palermo, Italy

**Keywords:** breast cancer stem cells, chemokines, CXCL1, tumor microenvironment, immunity genes, triple-negative breast cancer

## Abstract

Breast cancer (BC) mortality is mainly due to metastatic disease, which is primarily driven by cancer stem cells (CSC). The chemokine C-X-C motif ligand-1 (CXCL1) is involved in BC metastasis, but the question of whether it regulates breast cancer stem cell (BCSC) behavior is yet to be explored. Here, we demonstrate that BCSCs express CXCR2 and produce CXCL1, which stimulates their proliferation and self-renewal, and that CXCL1 blockade inhibits both BCSC proliferation and mammosphere formation efficiency. CXCL1 amplifies its own production and remarkably induces both tumor-promoting and immunosuppressive factors, including *SPP1/OPN*, *ACKR3/CXCR7*, *TLR4*, *TNFSF10/TRAIL* and *CCL18* and, to a lesser extent, immunostimulatory cytokines, including *IL15*, while it downregulates *CCL2*, *CCL28*, and *CXCR4*. CXCL1 downregulates *TWIST2* and *SNAI2*, while it boosts *TWIST1* expression in association with the loss of E-Cadherin, ultimately promoting BCSC epithelial-mesenchymal transition. Bioinformatic analyses of transcriptional data obtained from BC samples of 1,084 patients, reveals that *CXCL1* expressing BCs mostly belong to the Triple-Negative (TN) subtype, and that BC expression of *CXCL1* strongly correlates with that of pro-angiogenic and cancer promoting genes, such as *CXCL2-3-5-6*, *FGFBP1*, *BCL11A*, *PI3*, *B3GNT5*, *BBOX1*, and *PTX3*, suggesting that the CXCL1 signaling cascade is part of a broader tumor-promoting signaling network. Our findings reveal that CXCL1 functions as an autocrine growth factor for BCSCs and elicits primarily tumor progression and immune escape programs. Targeting the CXCL1/CXCR2 axis could restrain the BCSC compartment and improve the treatment of aggressive BC.

## Introduction

Breast cancer (BC) is the second most common cause of death from cancer in women (Sung et al., 2021). BC related deaths are mainly due to disease progression or recurrences, which are estimated to range between 20 and 30% of all BC cases (DeSantis et al., 2019). Moreover, approximately 6–10% of newly diagnosed BCs are initially stage IV or metastatic (DeSantis et al., 2019). Cancer stem cells (CSC), endowed with high plasticity and self-renewal properties, are the driving force of cancer progression and metastasis (Dittmer, 2018). Their quiescent, slow cycling, state accounts for chemo- and radiotherapy resistance (De Angelis et al., 2019), while their exit from dormancy and cell cycle re-activation, which precedes spreading and proliferation to distant organs, accounts for cancer relapse (De Angelis et al., 2019). The transition between these two CSC states is tightly regulated by cell-intrinsic mechanisms, systemic factors and interactions with the microenvironment, such as those mediated by immunoregulatory messengers (Prager et al., 2019).

Chemokines are soluble, small molecular weight (8–14 kDa) immunoregulatory proteins, which are essential for immune cell homing and play a key role in inflammation, host defense, angiogenesis, wound healing, but also in tumorigenesis and cancer immunoediting (Griffith et al., 2014; Nagarsheth et al., 2017).

The chemokine C-X-C motif ligand 1 (CXCL1), also named GROα, signals through the G protein-coupled receptor, C-X-C motif chemokine receptor 2 (CXCR2), to promote angiogenesis (Strieter et al., 2005) and to attract and activate neutrophils and basophils during inflammation (Baggiolini et al., 1994; Clark-Lewis et al., 1995).

Growing evidence supports a role for CXCL1 in cancer progression and recurrence. Originally identified as a melanoma growth stimulatory activity protein (Dhawan and Richmond, 2002), CXCL1 is constitutively highly expressed in melanoma cells and cooperates with oncogenic drivers, or loss of tumor suppressors, to promote tumor development (Luan et al., 1997).

In gastric cancer, overexpression of the CXCL1–CXCR2 axis is closely associated with the migration and invasiveness of malignant cells (Cheng et al., 2011), and CXCL1 release by the lymphatic endothelium promotes lymph node metastasis (Wang Z. et al., 2017).

In bladder cancer patients, urinary CXCL1 can serve as a molecular marker for tumor detection and as a predictor of local recurrence (Kawanishi et al., 2008; Nakashima et al., 2015).

In castration-resistant prostate cancer (Shamaladevi et al., 2009), overexpression of *CXCL1* promotes cancer cell epithelial-mesenchymal transition (EMT) and invasiveness, *via* AKT/NF-κB signaling pathway, thus favoring tumor progression (Kuo et al., 2012).

In colorectal cancer, a high level of *CXCL1* expression correlates with advanced tumor stage, shorter overall survival (OS) and disease-free survival (Wen et al., 2006; Zhuo et al., 2018).

In BC, expression of *CXCL1* is elicited by chemotherapy and promotes intratumoral recruitment of myeloid cells, which release chemokines that support BC cell survival and metastasis (Acharyya et al., 2012).

Although CXCL1 has been shown to be involved in BC progression and chemotherapy resistance (Minn et al., 2005; Acharyya et al., 2012), the question of whether it has a role in breast cancer stem cell (BCSC) behavior, which is the cornerstone of metastasis and resistance to chemotherapy, has never been addressed.

This study provides evidence that BCSCs produce and release CXCL1 and respond to the chemokine, which affects their viability and shapes their transcriptional profile. Bioinformatic analyses of microarray data, provided by the *TCGA PanCancer collection*, highlight the clinico-pathological relevance of our findings and reveal that expression of CXCL1 is prevalent in Triple-Negative (TN) BC, the molecular subtype with the highest CSC content and the worst clinical outcome (Honeth et al., 2008; Ma et al., 2014). Uncovering key pathways of BC progression is essential for the development of successful metastasis prevention and treatment strategies.

## Materials and Methods

### Cell Cultures and MTT Cell Proliferation Assays

The human (h) BC cell lines, namely BCSC-105 and BCSC-608, were generated and provided by Prof. G. Stassi (University of Palermo, Italy), who characterized them as BC stem cells (Todaro et al., 2013). BCSCs were authenticated by surface staining for characteristic markers, as described (Todaro et al., 2013). The culture medium consisted of serum-free DMEM:F12 (1:1), enriched with GlutaMAX-I supplement (Thermo Fisher Scientific, Waltham, MA, United States), 50 ng/ml heparin (Sigma-Aldrich, St. Louis, MO, United States), 20 ng/ml EGF, 10 ng/ml βFGF (R&D Systems, Minneapolis, MN, United States), and free from proteins, lipids, or growth factors.

Cell proliferation was assessed using the CellTiter 96^®^ AQueous One Solution Cell Proliferation Assay (Promega, Madison, WI, United States), according to manufacturer’s instructions. Briefly, cells were seeded on a 96-well plate, at a density of 1 × 10^3^ cells per well, and incubated, for 48 h, with recombinant (r) hCXCL1 (#300-11, Peprotech, Cranbury, NJ, United States), at concentration of 5, 10, 30, and 50 ng/ml, or with neutralizing anti-CXCL1 Ab (R&D Systems Cat# AF275, RRID:AB_355288), at concentration of 0.5, 1, 2, 5 μg/ml, for BCSC-105, and 0.030, 0.075, 0.150, 0.300 μg/ml, for BCSC-608.

Then, the plates were read at 490 nm, using the SpectraMax 190 microplate reader (RRID:SCR_018932; Molecular Devices San Jose, CA, United States). The proliferation was measured using untreated control cells as reference and the results were given as mean ± SD of three independent experiments carried out in triplicate.

### Flow Cytometry

To assess phenotype markers, BCSCs were harvested and mechanically dissociated into a single cell suspension. Then, the cells were pelleted, resuspended in PBS and incubated for 30 min, at 4°C, with the following antibodies (Abs): anti-CD24 (RRID:AB_10562033), anti-CD29 (RRID:AB_395836), anti-CD44 (RRID:AB_398683), and anti-CD117 (RRID:AB_398461) (all from BD Biosciences, Franklin Lakes, NJ, United States), anti-CD133 (RRID:AB_2726287) (Miltenyi Biotec, Bergisch Gladbach, Germany), and anti-CXCR2 (RRID:AB_2296102) (R&D Systems, Minneapolis, MN, United States), at a concentration of 0.25 μg/100μl. Acquisition was performed using a BD Scientific Canto II Flow Cytometer (RRID:SCR_018056) and the data were analyzed using FlowJo software (FlowJo, RRID:SCR_008520). Dead cells were excluded by 7AAD staining. All experiments were performed in triplicate.

### ELISA

Assessment of CXCL1 protein in the supernatant derived from BCSCs was performed using the GRO alpha (CXCL1) Human ELISA Kit (BMS2122, Thermo Fisher Scientific, Waltham, MA, United States; detection sensitivity: 0.7–7.6 pg/ml), according to manufacturer’s instructions, using the same culture conditions described above.

### Sphere Formation Assay

The sphere-forming potential of BCSC-105 and BCSC-608 was assessed by using the Extreme Limiting Dilution Analysis (ELDA, RRID:SCR_018933) (Hu and Smyth, 2009).

Briefly, cells were seeded at concentrations of one cell, two cells, four cells, or eight cells per well, on 96-well ultra-low attachment plates, and incubated at 37°C, with 5% CO_2,_ in a humidified incubator for 8 days, with or without rhCXCL1 (Peprotech, London, United Kingdom; 10 ng/ml for BCSC-105 and 30 ng/ml for BCSC-608) or anti-CXCL1 Ab (R&D Systems, Minneapolis, MN, United States; 5 μg/ml for BCSC-105 and 0.15 μg/ml for BCSC-608).

Spheres containing ≥ 3 cells were counted under a Leica light microscope.

### Histopathology and Immunohistochemistry

Histology and immunohistochemistry on formalin fixed paraffin embedded human TNBC samples, were performed as reported (Di Meo et al., 2014), by using anti-CXCL1 (R&D Systems Cat# AF275, RRID:AB_355288), anti-CD68 (Agilent Cat# M0876, RRID:AB_2074844), and anti-CD133 Abs (Cell Signaling Technology Cat# 3663, RRID:AB_2172866). Written informed consent was obtained from patients. The study was performed in accordance with the principles outlined in the Declaration of Helsinki and approved by the Ethical Committee of the “G. d’Annunzio” University and Local Health Authority of Chieti, Italy (Protocol ONCO-2017-1, 04/19/2018).

### Immunofluorescence Staining and Confocal Microscopy

Immunofluorescent stainings of 4% PFA-fixed BCSCs were performed as follows: primary Abs goat anti-CXCL1 (R&D Systems Cat# AF275, RRID:AB_355288), rabbit anti-OPN (Abcam Cat# ab14175, RRID:AB_2194831), goat anti-TRAIL (Santa Cruz Biotechnology Cat# sc-6079, RRID:AB_2205918), and mouse anti-TWIST1 (Abcam Cat# ab50887, RRID:AB_883294) were incubated overnight at 4°C, followed by incubation with Alexa Fluor 633 anti-goat IgG (Thermo Fisher Scientific Cat# A-21082, RRID:AB_2535739), Alexa Fluor 488 anti-rabbit IgG (Thermo Fisher Scientific Cat# A32731, RRID:AB_2633280), or Alexa Fluor 594 anti-mouse IgG (Molecular Probes Cat# A-11005, RRID:AB_141372) for 2 h at room temperature.

For double immunofluorescent stainings, slides were incubated, for 3 h at room temperature, with mouse anti-TWIST1 Ab (Abcam Cat# ab50887, RRID:AB_883294), followed by incubation with Alexa Fluor 594 anti-mouse IgG (Molecular Probes Cat# A-11005, RRID:AB_141372), for 2 h at room temperature. Then, slides were incubated, overnight at 4°C, with anti-E-cadherin Ab (Agilent Cat# M3612, RRID:AB_2076672), followed by incubation with Oregon Green 488 anti-mouse IgG (Thermo Fisher Scientific Cat# O-11033, RRID:AB_2539797), for 2 h at room temperature.

Nuclei were counterstained with 4′,6-Diamidino-2-Phenylindole, Dihydrochloride (DAPI) (#D1306, Thermo Fisher Scientific, Waltham, MA, United States) for 2 min and slides were analyzed under an LSM 510 Meta confocal microscope (Zeiss, Oberkochen, Germany; RRID:SCR_018062).

### PCR Array and Real-Time RT-PCR

RNA was extracted by using the RNeasy Mini Kit (#74104, Qiagen, Hilden, Germany), and reverse-transcribed with the RT2 First Strand Kit (#33040, Qiagen, Hilden, Germany). PCR array analyses were run on a Qiagen Rotor Gene Q (Qiagen Rotor-Gene Q, RRID:SCR_018976), using the RT^2^ Profiler Human Cancer Inflammation and Immunity Crosstalk PCR Array (#PAHS-181Z, Qiagen, Hilden, Germany) and RT2 SYBR Green ROX Fast Master mix (#330623, Qiagen, Hilden, Germany). The results from each plate were normalized to the median value of a set of housekeeping genes. Changes in the gene expression were calculated using the ΔΔCt method. Results from experiments were performed in triplicate, pooled and analyzed with the manufacturer’s software. A significant threshold of a twofold change in gene expression corresponded to a *p* < 0.001.

For single gene analyses, real-time RT-PCR was performed using the Quantifast SYBR Green PCR Kit (#204054, Qiagen, Hilden, Germany) and a MiniOpticon System (#CFB-3120, Bio-Rad, Hercules, CA, United States).

Primers for housekeeping gene *hypoxanthine phosphoribosyltransferase* (*HPRT*), *MMP2, MMP9, OCT4A*, *SNAI1, SNAI2, TWIST1, TWIST2, YAP1, ZEB1, ZEB2* were designed and synthesized by Sigma-Aldrich Corporation (St. Louis, MO, United States): *HPRT* Forward 5′- AGA CTTTGCTTTCCTTGGTCAGG-3′ and *HPRT* Reverse 5′-GTCTGGCTTA TATCCAACACTTCG-3′; *MMP2* Forward 5′-AGCGAGTGGATGCCGCCTTTAA-3′ and *MMP2* Reverse 5′-CATTCCAGGCATCTGCGATGAG-3′; *MMP9* Forward 5′-GCC ACTACTGTGCCTTTGAGTC-3′ and *MMP9* Reverse 5′-CCCTCAGAGAATCGCCAGTACT-3′; *OCT4A* Forward 5′-CCCCTGGTGCCGTGA-3′ and *OCT4A* Reverse 5′-GCAAATTGCTCGAGTTCTTTCTG-3′; *SNAI1* Forward 5′-CCTCTTCCTCTCCATACCT-3′ and *SNAI1* Reverse 5′-TTC ATCAAAGTCCTGTGGG-3′; *SNAI2* Forward 5′-TGT CATACCACAACCAGAGA-3′ and *SNAI2* Reverse 5′-CTTGGAGGAGGTGTCAGAT-3′; *SOX2* Forward 5′-AGA GAGAAAGAAAGGGAGAGA-3′ and *SOX2* Reverse 5′-AAT CAGGCGAAGAATAATTTGG-3′; *TWIST1* Forward 5′-CGG AGACCTAGATGTCATT-3′ and *TWIST1* Reverse 5′-CTGTCTCGCTTTCTCTTTT-3′; *TWIST2* Forward 5′-AACTGGACCAAGGCTCTC-3′ and *TWIST2* Reverse 5′-GCGGCGTGAAAGTAAGAAT-3′; *YAP1* Forward 5′-TTCCTCTCCAGCTTCTCTGC-3′ and *YAP1* Reverse 5′-GATGCTGAGCTGTGGGTGTA; *ZEB1* Forward 5′-CCAACAGACCAGACAGTG-3′ and *ZEB1* Reverse 5′-TGACTCGCATTCATCATCTT; *ZEB2* Forward 5′-CGGAGACTTCAAGGTATAATCTATC-3′ and *ZEB2* Reverse 5′-GTTACGCCTCTTCTAATGACAT-3′. Primers for *BMI1* (#QT00052654), *KLF4* (#QT00061033), *MET* (#QT00023408), *MMP14* (#QT00001533), *MYC* (#QT00035406), *NOTCH1* (#QT00231056), *SHH* (#QT01156799) and *WWTR1* (#QT01017996) were purchased from Qiagen, Hilden, Germany.

Melting curve analysis was done to assess the specificity of PCR products and the efficiency of reaction for each target was evaluated by amplifying serial dilutions of cDNA. Relative quantification of mRNA was done according to the comparative threshold cycle method with HPRT as calibrator, using the Bio-Rad CFX Manager software. The samples were processed in triplicate, and wells without added cDNA served as negative controls.

Pooled results ± SD are from two experiments performed in duplicate. A significant threshold of fourfold change in gene expression corresponded to *p* < 0.001, and only genes above the threshold, in both cell lines, are represented.

### Bioinformatic Analyses

For bioinformatic analyses (cBioPortal, RRID:SCR_014555), gene expression data from the “*Breast Invasive Carcinoma TCGA PanCancer collection*” dataset (Berger et al., 2018), which includes 1,084 BC cases, were downloaded from the cBioportal for Cancer Genomics database (^1^ cBioPortal, RRID:SCR_014555). For each sample, the Z-scores of *CXCL1*mRNA levels were calculated, compared to the mean of all samples in the study, and all samples with a Z-score ≥ 2 were considered *CXCL1*-expressing. Subsequently, the association between *CXCL1*mRNA expression and BC subtypes was assessed using Fisher’s exact test, whereas the correlation between *CXCL1*mRNA expression and the expression of other genes was assessed using the Spearman’s correlation coefficient (ρ). All statistical tests were evaluated at an α level of 0.05.

### Statistical Analyses

For *in vitro* studies, between-group differences were assessed by Student’s *t*-test or ANOVA (followed by Tukey’s HSD test). Between groups differences in sphere-forming potential were evaluated by ELDA (Hu and Smyth, 2009). All statistical tests were evaluated at an α level of 0.05, using Stata, version 13 (StataCorp, College Station, TX, United States; RRID:SCR_012763).

## Results

### CXCL1 Autocrine Signaling Sustains BCSC Proliferation and Mammosphere Formation Efficiency

Human CSCs, BCSC-105 and BCSC-608, were isolated from distinct infiltrating ductal BCs with different genetic and molecular background, and showed a CD133^+^CD44^+^CD24^*low*^ phenotype (Todaro et al., 2013; Sorrentino et al., 2021, submitted). BCSCs fulfilled the functional properties of CSCs, such as the ability to grow in tumor spheres and to reproduce the histological and immunophenotipical features of the tumor of origin, when implanted, at low cell numbers, in immunocompromised mice (Todaro et al., 2013).

**FIGURE 1:**
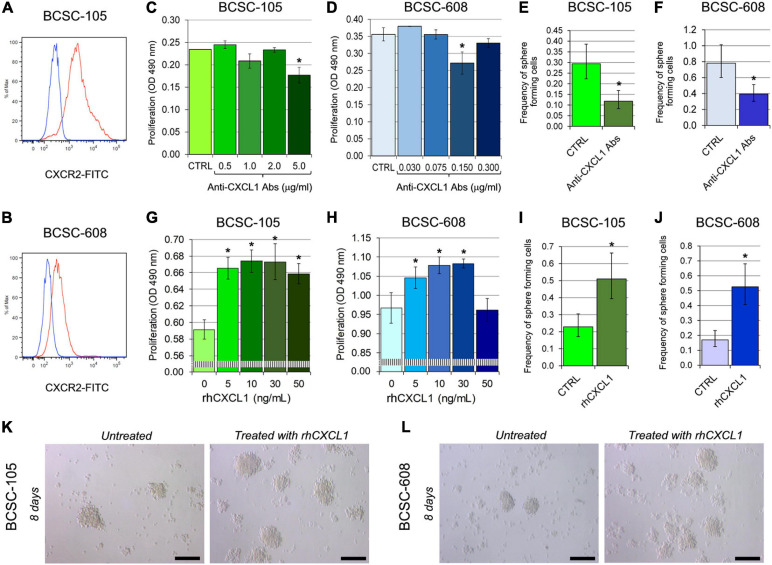
CXCL1 regulates the proliferation and self-renewal ability of BCSCs. **(A,B)** Flow cytometry analysis of CXCR2 expression in **(A)** BCSC-105 and **(B)** BCSC-608. Red profiles illustrate the expression of CXCR2, while blue profiles represent isotype controls. Each panel is representative of three independent experiments. **(C)** MTT assay of BCSC-105 cells treated with different concentrations of anti-CXCL1 Abs. CTRL: untreated cells. ANOVA: *p* = 0.0002. *p < 0.05, Tukey HSD Test compared with CTRL, 0.5, 1.0, 2.0, and 5.0 μg/ml. Experiments were performed in triplicate. **(D)** MTT assay of BCSC-608 cells treated with different concentrations of anti-CXCL1 Abs. CTRL: untreated cells. ANOVA: *p* = 0.0022. *p < 0.05, Tukey HSD Test compared with CTRL, 0.030, 0.075, 0.150, and 0.300 μg/ml. Experiments were performed in triplicate. **(E)** Sphere forming capability of BCSC-105, evaluated by ELDA, after 8 days of treatment with 5.0 μg/ml of anti-CXCL1 Abs. *p = 0.0001, Chi-squared test compared with untreated cells (CTRL). Experiments were performed in triplicate. **(F)** Sphere forming capability of BCSC-608, evaluated by ELDA, after 8 days of treatment with 0.15 μg/ml of anti-CXCL1 Abs. *p = 0.0002, Chi-squared test compared with untreated cells (CTRL). Experiments were performed in triplicate. **(G)** MTT assay of BCSC-105 stimulated with different concentrations of rhCXCL1. ANOVA: *p* < 0.001. *p < 0.01, Tukey HSD Test compared with 0 ng/ml. **(H)** MTT assay of BCSC-608 stimulated with different concentrations of rhCXCL1. ANOVA: *p* < 0.001. *p < 0.01, Tukey HSD Test compared with 0 ng/ml. **(I)** Sphere forming capability of BCSC-105, evaluated by ELDA, after 8 days of treatment with 10 ng/ml of rhCXCL1. *p < 0.0001, Chi-squared test compared with untreated cells (CTRL). Experiments were performed in triplicate. **(J)** Sphere forming capability of BCSC-608, evaluated by ELDA, after 8 days of treatment with 30 ng/ml of rhCXCL1. *p < 0.0001, Chi-squared test compared with untreated cells (CTRL). Experiments were performed in triplicate. **(K)** BCSC-105 and **(L)** BCSC-608–derived spheres were dissociated, seeded at concentrations of 1 cell/well, and untreated or treated with 10 and 30 ng/ml of rhCXCL1, respectively. Magnification: X400. Scale bars: 100 μm.

To investigate whether BCSCs are responsive to CXCL1, we first analyzed, by flow cytometry, the expression of its cognate receptor CXCR2. Both BCSC-105 and BCSC-608 expressed CXCR2 (Figures 1A,B) and constitutively produced and released CXCL1, specifically, 155.07 and 3.33 pg/ml, respectively. The addition of anti-CXCL1 neutralizing Abs to the culture medium of BCSC-105 and BCSC-608 significantly inhibited their proliferation (BCSC-105: ANOVA, *p* = 0.0002; BCSC-608: ANOVA, *p* = 0.0022) (Figures 1C,D) and mammosphere-formation efficiency (BCSC-105: Chi-squared test, *p* = 0.0001; BCSC-608: Chi-squared test, *p* = 0.0002, Figures 1E,F). By contrast, treatment with rhCXCL1 (5–50 ng/ml for 48 h) increased the proliferation of BCSCs (ANOVA, *p* < 0.001; Figures 1G,H), and boosted their mammosphere formation ability (Chi-squared test, *p* < 0.0001; Figures 1I–L).

### CXCL1 Shapes Immune Gene Expression Profile of BCSCs and Promotes Tumor Progression and Immune Evasion Programs

**FIGURE 2:**
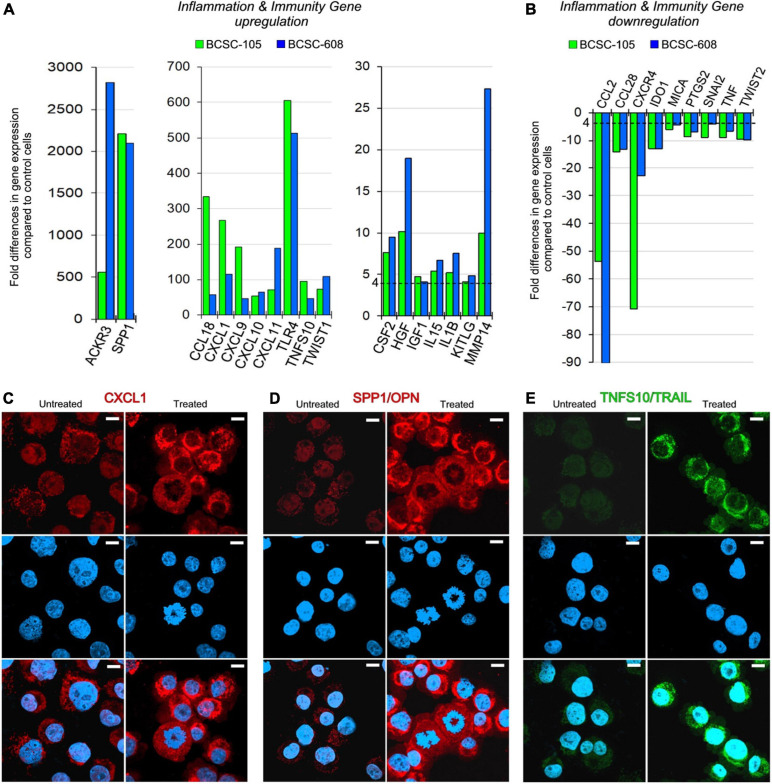
CXCL1 upregulates its own expression and shapes the transcriptional profile of BCSCs. **(A,B)** Fold differences in mRNAs of inflammation and immunity genes expression [upregulated genes are represented in panel **(A)**, and downregulated genes are represented in panel **(B)**] between rhCXCL1 treated and untreated BCSC-105 (green bars) and BCSC-608 (blue bars). Pooled results ± SD are from two experiments performed in duplicate. A significant threshold of a fourfold change in gene expression corresponded to *p* < 0.001. **(C)** Confocal microscopy images of CXCL1 (red) in untreated and rhCXCL1 treated BCSC-105 cells. DAPI-DNA stained nuclei. Magnification: X630. Scale bars: 3 μm. **(D)** Confocal microscopy images of OPN (red) in untreated and rhCXCL1 treated BCSC-105 cells. DAPI-DNA stained nuclei. Magnification: X630. Scale bars: 3 μm. **(E)** Confocal microscopy images of TRAIL (green) in untreated and rhCXCL1 treated BCSC-105 cells. DAPI-DNA stained nuclei. Magnification: X630. Scale bars: 3 μm.

In both BCSC-105 and BCSC-608, treatment with rhCXCL1 (10 ng/ml) considerably amplified its own expression (267.19 times in BCSC-105; 115.53 times in BCSC-608; Figures 2A,C), and strongly promoted the expression of *ACKR3/CXCR7* (560.94 times in BCSC-105; 2,820.98 times in BCSC-608), and *SPP1/OPN* (2,212.88 times in BCSC-105; 2,094.11 times in BCSC-608; Figures 2A,D).

Treatment of hBCSCs with rhCXCL1 also increased the expression of cytokines, chemokines, chemokine receptors, growth factors and metalloproteinase, in particular, *IL1*β (5.25 times in BCSC-105; 7.53 times in BCSC-608), *IL15* (5.40 times in BCSC-105; 6.74 times in BCSC-608), *CCL18* (333.54 times in BCSC-105; 58.17 times in BCSC-608), *CXCL9* (192.03 times in BCSC-105; 46.60 times in BCSC-608), *CXCL10* (53.88 times in BCSC-105; 64.54 times in BCSC-608), *CXCL11* (71.10 times in BCSC-105; 187.89 times in BCSC-608), *CSF2* (7.63 times in BCSC-105; 9.53 times in BCSC-608), *IGF1* (4.09 times in BCSC-608; 4.80 times in BCSC-105), *HGF* (10.17 times in BCSC-105; 18.97 times in BCSC-608) and *MMP14* (9.95 times in BCSC-105; 27.34 times in BCSC-608) (Figure 2A).

The expression of specific surface molecules, which regulate immune evasion mechanisms (Koyama et al., 2002; Sato et al., 2009; Kuonen et al., 2012) was also significantly increased. In particular, the treatment with rhCXCL1 stimulated the expression of *TLR4* (605.39 and 512.75 times, in BCSC-105 and BCSC-608), of *KITLG* (4.10 and 4.86 times, in BCSC-105 and BCSC-608), and of *TNFS10/TRAIL* (94.47 and 47.25 times, in BCSC-105 and BCSC-608; Figures 2A,E).

**FIGURE 3:**
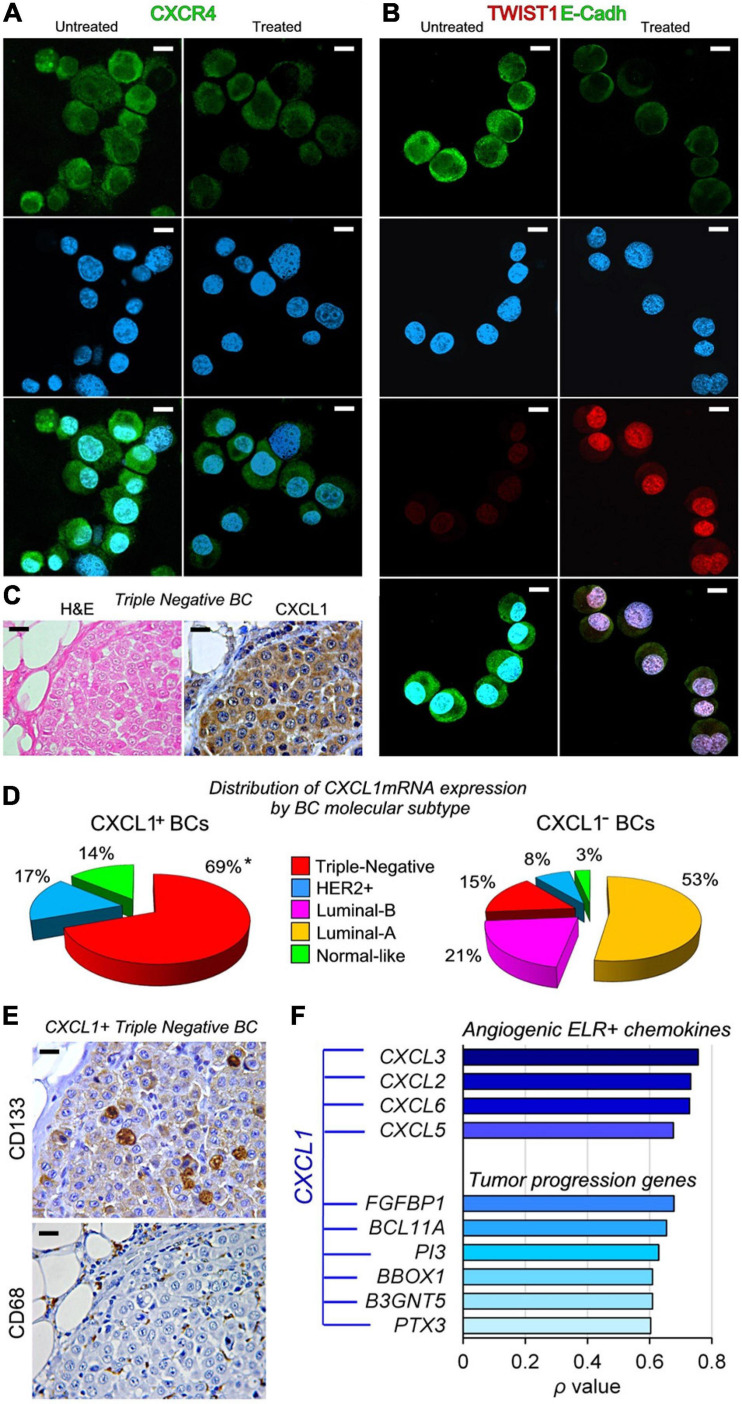
CXCL1 has a dual role in regulating tumor progression genes in BCSCs and is primarily expressed in Triple-Negative BC molecular subtype. **(A)** Confocal microscopy images of CXCR4 (green) in untreated and rhCXCL1 treated BCSC-105 cells. DAPI-DNA stained nuclei. Magnification: X630. Scale bars: 3 μm. **(B)** Confocal microscopy images of TWIST1 (red) and E-Cadherin (green) in untreated and rhCXCL1 treated BCSC-105 cells. DAPI-DNA stained nuclei. Magnification: X630. Scale bars: 3 μm. **(C)** Hematoxylin and eosin (H&E) staining and immunostaining with anti-CXCL1 Abs of a representative Triple-Negative BC sample. Magnification: X400. Scale bars: 20 μm. **(D)** Distribution of CXCL1^+^BCs and CXCL1^−^ BCs by molecular subtypes, represented as percentage of the total number of BC expressing or not *CXCL1*mRNA. *p < 0.0001, Chi-squared test vs Triple-Negative CXCL1^−^BCs. **(E)** Immunostaining with anti-CD133 stem cell marker and anti-CD68 Abs of a representative CXCL1^+^Triple-Negative BC sample. Magnification: X400. Scale bars: 20 μm. **(F)** Correlation between the expression of *CXCL1mRNA* in human BC samples (from the “*Breast Invasive Carcinoma TCGA PanCancer collection”)* and that of angiogenic ELR^+^ chemokines and tumor progression genes, measured by Spearman’s rank correlation coefficient (ρ). Strength of the Correlation: 0.00 ≤ ρ ≤ 0.19, very weak; 0.20 ≤ ρ ≤ 0.39, weak; 0.40 ≤ ρ ≤ 0.59, moderate; 0.60 ≤ ρ ≤ 0.79, strong; 0.80 ≤ ρ ≤ 1.0, very strong.

By contrast, the treatment of BCSCs with rhCXCL1 inhibited their expression of *CCL2* (−53.59 and −90.00 times in BCSC-105 and BCSC-608), *CCL28* (−14.20 and −13.16 times in BCSC-605 and BCSC-608), *CXCR4* (−70.89 and −22.75 times, in BCSC-105 and BCSC -608) (Figures 2B, 3A), *TNF* (−8.99 and −6.58 times, in BCSC-105 and BCSC-608), *IDO1* (−12.89 and −13.07 times, in BCSC-105 and BCSC-608), *PTGS2* (−8.62 and −7.10 times, in BCSC-105 and BCSC-608) and *MICA/MHCI* (−6.14 and −4.40 times, in BCSC-105 and BCSC-608) (Figure 2B).

Analyses of genes that regulate stemness (*SHH, OCT4, SOX2, KLF4, NOTCH, MYC, YAP*, and *WWTR1*), and EMT, (*SNAI1, SNAI2, ZEB1, ZEB2, TWIST1, TWIST2*, and *MET*), showed that CXCL1 downregulated *TWIST2* and *SNAI2* (−9.69 and −9.10 times in BCSC-105, and −9.89 and −4.05 times in BCSC-608, respectively; Figure 2B), but strongly stimulated BCSC expression of *TWIST1* (73.38 times in BCSC-105; 108.57 times in BCSC-608) in association with a distinct loss of E-Cadherin expression (Figures 2A, 3B).

### Expression of CXCL1 Is Prevalent in Triple-Negative BC and Positively Correlates with the Expression of Pro-Angiogenic Factors and Tumor Progression Genes

Previous studies have revealed that *CXCL1* expression is correlated with overall survival (OS) and relapse-free survival (RFS) in BC patients, and is predictive of a poor prognosis (Divella et al., 2013; Zou et al., 2014). The growth factor activity of CXCL1 in BCSCs led us to assess the extent of its expression in tumor tissues from BC patients in order to determine its clinic-pathological impact.

Bioinformatic analyses of gene expression data obtained from whole tumor samples of 1,084 BC patients included in the “*Breast Invasive Carcinoma TCGA PanCancer collection*” dataset (Berger et al., 2018), identified the expression of *CXCL1mRNA* in 3.67% of BCs, and its association with the TNBC subtype (Chi-squared test: *p* < 0.0001; Figure 3C). As represented in Figure 3D, 69% of CXCL1^+^BCs, and only 15% of CXCL1^–^BCs, were diagnosed as TNBC.

Immunohistochemistry revealed that, in CXCL1^+^TNBCs, the cellular sources of CXCL1 included, in addition to CD133^+^BCSCs (Park et al., 2010), tumor infiltrating immune cells, such as CD68^+^ macrophages (Figure 3E), and the vast majority of BC cells.

Irrespective of the molecular subtype of BC, analyses of microarray data also revealed, a strong positive correlation between the expression of *CXCL1mRNA* in BC samples, and that of angiogenic ELR^+^ chemokines, such as *CXCL3* (ρ = 0.756), *CXCL2* (ρ = 0.732), *CXCL6* (ρ = 0.728), *CXCL5* (ρ = 0.676), and tumor progression genes, specifically, *FGFBP1* (ρ = 0.678), *BCL11A* (ρ = 0.654), *PI3* (ρ = 0.629), *B3GNT5* (ρ = 0.609), *BBOX1* (ρ = 0.609), and *PTX3* (ρ = 0.603). This finding suggests that the CXCL1 signaling pathway is part of a broader BC progression program with important clinical implications (Figure 3F).

## Discussion

Overcoming the challenge of metastatic BC is a major public health issue, as it causes about 630,000 deaths worldwide each year (Sung et al., 2021). Understanding the molecular pathways regulating BCSCs, which are the driving force of metastasis, is critical to achieve this goal.

Here we demonstrate that CSCs, derived from BCs with different genetic and molecular background (Todaro et al., 2013), reveal different levels of production and responsiveness to CXCL1 that acts as an autocrine growth factor for BCSCs, regardless of the BC subtype from which they originate. BCSCs constitutively release and respond to the chemokine, which sustains their proliferation and self-renewal, and reshapes their transcriptional profile, ultimately promoting tumor invasion and immune evasion programs.

The ELR^+^ chemokine CXCL1 signals through CXCR2 to promote angiogenesis and regulates host immune response, by recruiting and activating neutrophils and basophils during inflammation (Baggiolini et al., 1994; Clark-Lewis et al., 1995; Strieter et al., 2005).

In the inflammatory tumor microenvironment, macrophages, myeloid derived suppressor cells (MDSC), endothelial cells and stromal fibroblasts secrete CXCL1, which activates the NF-kB pathway in CXCR2^+^BC cells (Wang et al., 2018) and promotes tumor growth, while contributing to local immunosuppression. CXCL1, produced in the primary tumor, recruits MDSCs to form the pre-metastatic niche, sustaining homing, survival, and growth of circulating tumor cells in secondary organs leading to metastasis development (Wang D. et al., 2017).

Although bioinformatics reveal that only 3.67% of BCs, from different stages of disease, included in the PanCancer collection, express *CXCL1*, the high incidence of this tumor, estimated at 2,000,000 cases per year worldwide, means that ∼73,000 patients are expected to be diagnosed with CXCL1^+^BC each year.

**FIGURE 4:**
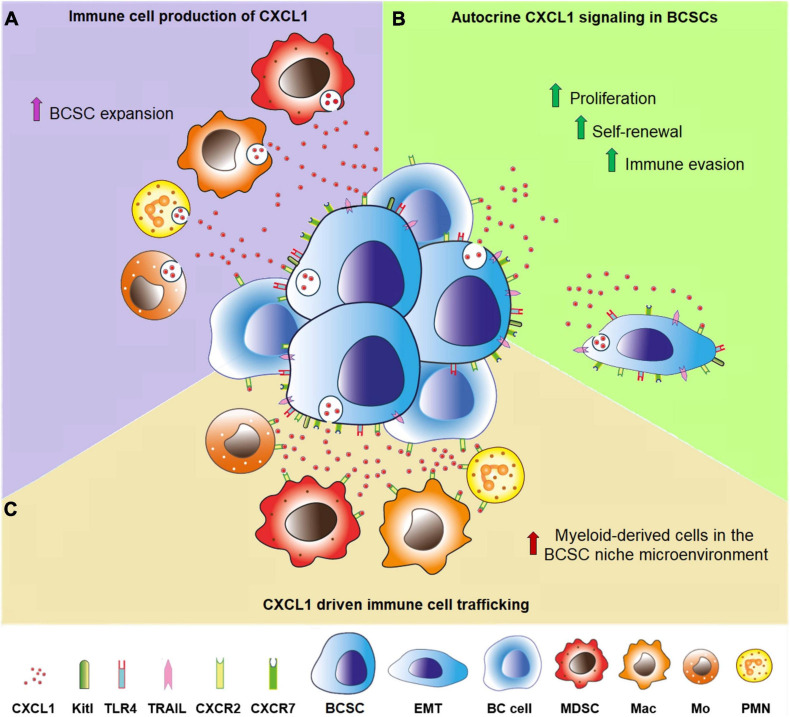
CXCL1 production in the BCSC niche microenvironment. **(A)** Purple room. Polymorphonuclear neutrophils (PMN), myeloid-derived suppressor cells (MDSC), monocytes (Mo) and macrophages (Mac) produce and release CXCL1, which regulates the behavior of BCSCs and BC cells endowed with CXCR2. **(B)** Green room. CXCL1 secreted by BCSCs fosters, *via* autocrine and paracrine signaling, their proliferation and self-renewal, promotes their epithelial-mesenchimal transition (EMT) and immune evasion, by the induction of Kitl, TLR4, TRAIL, and regulates the behavior of the surrounding BC cells. **(C)** Brown room. CXCL1 secreted by BCSCs regulates immune cell trafficking, especially by recruiting polymorphonuclear neutrophils, monocytes, macrophages and myeloid-derived suppressor cells.

Our study adds a new piece to the puzzle of the BC microenvironment (Figure 4), by revealing that BCSCs can be a prominent source of CXCL1, particularly in TNBC, which is enriched in CSCs (Honeth et al., 2008; Ma et al., 2014) and more frequently expresses *CXCL1mRNA*, as evidenced by bioinformatic analyses of data obtained from the PanCancer database (Berger et al., 2018). Defined by the lack of ER, PR, and HER2 expression, TNBC represents about 15–20% of invasive BCs and is associated with high risk of metastasis and chemotherapy resistance (Dai et al., 2015). *CXCL1* expression likely contributes to this malignant phenotype, since 69% of patients bearing CXCL1^+^BCs, were diagnosed as TNBCs.

In addition to the constitutive production by BCSCs, and possibly other BC cells and stromal mesenchymal stem cells (Wang Y. et al., 2017), CXCL1 can be dynamically induced, during tumor progression, in macrophages, MDSCs, granulocytes, endothelium and fibroblasts, by a variety of stimuli such as IL1β, TNFα (Wen et al., 1989; Acharyya et al., 2012), IL6 (Roy et al., 2012), PGE2 (Wang et al., 2006), adipokines (Wang et al., 2020), and TLR3/4 (Zhao et al., 2014).

In BCSCs, CXCL1 strengthens its own production and dramatically boosts *SPP1/OPN* and *ACKR3/CXCR7* expression. *Secreted Phosphoprotein 1 (SPP1)* gene encodes for *Osteopontin (OPN)*, a sialic acid rich, chemokine-like, matricellular phosphoglycoprotein, with well-defined roles in cell-matrix interaction, inflammatory responses, angiogenesis, and tumor metastasis (Shevde and Samant, 2014). OPN regulates the expression of genes leading to multiple signal transduction events associated with BC growth and progression (Cook et al., 2005). *ACKR3* gene encodes for *CXCR7*, the receptor for CXCL11 and CXCL12/SDF1, that promotes cell proliferation and invasive migration (Miao et al., 2007; Stacer et al., 2016), and has proven to be crucial for BCSC tumorigenicity and maintenance of stemness properties (Tang et al., 2016).

The considerable CXCL1-induced up-regulation of *TLR4*, *TNFSF10/TRAIL*, and *KITLG* expression along with *MICA/MHCI* down-regulation, enable tumor evasion from immune surveillance.

TLR4 is a pattern recognition receptor (PRR) family member that confers the ability to “sense” damage signals, and activates innate immunity, that can amplify the tumor-associated inflammation (Medzhitov, 2001). Activated TLR signals on cancer cells promote their migration and induce immunosuppressive cytokines and apoptosis resistance (Sato et al., 2009; Yang et al., 2010). Invasiveness and angiogenetic potential of BC cells are supported by TLR4-mediated signaling pathways (Ahmed et al., 2013; Yang et al., 2014), leading to pro-tumoral effects, which may be TP53 dependent (Haricharan and Brown, 2015).

TNFSF10, also known as Tumor Necrosis Factor (TNF)-related apoptosis-inducing ligand (TRAIL) is a member of the TNF superfamily that triggers apoptosis by binding to death receptors, DR4 and DR5 (Falschlehner et al., 2007). While TRAIL expression on activated NK and T cells increases their cytotoxicity, CXCL1 induction of TRAIL on BCSCs can turn them into apoptosis inducers, which suppress neighbor cancer cells (Griffith et al., 2000; Koyama et al., 2002; Papageorgiou et al., 2004), but also T cell activation and proliferation, favoring tumor immune evasion (Inoue et al., 2002).

Expression of KIT Ligand, encoded by the *KITLG* gene, on BCSCs can lead to intratumoral recruitment of immunosuppressive KIT^+^CD11b^+^cells. Blocking of the KIT Ligand/KIT axis has been demonstrated to slow-down BC progression and metastasis (Kuonen et al., 2012).

MHC class I chain related-protein A (MICA) is a natural killer group 2D (NKG2D) ligand that triggers NK and Vδ1 γδ T cells and co-stimulates CD8αβ^+^T cells (Groh et al., 1998; Bauer et al., 1999). Reduced BCSC expression of MICA/MHCI is expected to weaken the cytolytic ability of effector cells and promote immune escape.

The tumor progression program triggered by CXCL1 in BCSCs also includes the expression of inflammatory mediators, proteases and growth factors, specifically, *IL1*β, *CCL18*, *CSF2, MMP14, HGF*, and *IGF1*. *IL1*β is involved in multiple aspects of tumor initiation and progression, and has shown to promote metastatic colonization of BCSCs to the bone (Mantovani et al., 2018; Eyre et al., 2019). CCL18 attracts naïve T cells, T regulatory cells, Th2 cells and immature dendritic cells (DC) (Adema et al., 1997; Chenivesse et al., 2012), and has been demonstrated to promote BC cell invasiveness and adherence to the extracellular matrix (Chen et al., 2011). CSF2/GM-CSF, produced by BC cells, activates plasmacytoid DCs leading to a regulatory Th2 response by naive CD4^+^T cells, which is associated with aggressive BC subtypes (Ghirelli et al., 2015). MMP14 sustains cancer cell trafficking through the extracellular matrix ECM (Rowe and Weiss, 2009), and strengthens BCSC ability in anchorage-independent growth, tumor initiation, invasion, and migration under hypoxic nutrient-deprived conditions (Hillebrand et al., 2019). Both HGF and IGF1 activated signaling pathways lead to BC cell proliferation, migration and invasion, and are critically involved in the induction/maintenance of EMT and cell stemness, which are fundamental in metastatic spread and resistance to anti-cancer treatments (Malaguarnera and Belfiore, 2014; Christopoulos et al., 2015; Owusu et al., 2017). Therefore, the EMT program activated by CXCL1 in BCSCs, and revealed by the loss of E-Cadherin and gain of a strong nuclear expression of TWIST1, likely results from a complex network of signaling pathways triggered by secondary mediators, that ultimately overwhelm the effects expected by CXCL1-dependent TWIST2 and SNAI2 down-regulation.

Intriguingly, while CXCL1 induces, by hundreds and thousands of times, tumor-promoting and immunosuppressive factors, it also promotes, though to a lesser extent, the expression of *IL15, CXCL9, CXCL10, CXCL11*, which can lead to T and NK cell recruitment and anti-tumor responses (Tagaya et al., 1996; Palacios-Arreola et al., 2014), and down-regulates *CCL2, CCL28, IDO, PTGS2, TNF* and *CXCR4*, that may inhibit inflammation and cancer cell migration (Müller et al., 2001; Ali and Lazennec, 2007; Howe, 2007). These findings reveal the unprecedented, apparently, dual role of CXCL1 in shaping the immunobiology of BCSCs, since it elicits a range of immunity genes with heterogeneous and opposing functions, including both pro- and anti-tumor mediators. Yet, the prominent expression of the former could explain why CXCL1 expression is associated with tumor progression (Divella et al., 2013; Zou et al., 2014) and with the highly malignant TNBC subtype.

The considerable CXCL1-dependent inhibition of the expression of CCL2 and CCL28, also endowed with an immunostimulating, but also pro-tumoral effect (Mohan et al., 2017; Yoshimura, 2018), emphasizes the critical role of the final equilibrium among the multiple microenvironmental signals in driving BCSC fate and tumor behavior.

Interestingly, independently of the molecular subtype, in BC the strong correlation between the expression of *CXCL1* and a range of pro-angiogenic and tumor promoting genes, including *CXCL3, CXCL2, CXCL6, CXCL5, FGFBP1* (Tassi et al., 2001; Strieter et al., 2005; Zheng et al., 2009), *BCL11A, PI3, B3GNT5, BBOX1* (Potapenko et al., 2010, 2015; Khaled et al., 2015; Pascual and Turner, 2019; Liao et al., 2020), and *PTX3* (Thomas et al., 2017; Zhang et al., 2020), suggests that CXCL1 regulated immunity genes are part of a wider signaling network that fuels BC progression.

Targeting CXCL1 signaling cascade, and closely associated pro-tumoral cues, could be a valuable strategy to restrain BCSC compartment and improve the efficacy of modern immunotherapeutic approaches to aggressive BCs.

## Data Availability Statement

The original contributions presented in the study are included in the article/supplementary material, further inquiries can be directed to the corresponding author/s.

## Ethics Statement

The studies involving human participants were reviewed and approved by Ethical Committee of the “G. d’Annunzio” University and Local Health Authority of Chieti, Italy. The patients/participants provided their written informed consent to participate in this study.

## Author Contributions

EDC conceived the study. SLC, LDA, CF, and PL performed the experiments, collected, and assembled the data. EDC, SLC, LDA, and CS performed data analyses. MT and GS provided cell lines for the study. EDC interpreted the data and wrote the manuscript. All authors contributed to manuscript revision and approved the submitted version.

## Conflict of Interest

The authors declare that the research was conducted in the absence of any commercial or financial relationships that could be construed as a potential conflict of interest.

## Footnotes

1 https://www.cbioportal.org
